# Effects of the Usage of l-Cysteine (l-Cys) on Human Health

**DOI:** 10.3390/molecules23030575

**Published:** 2018-03-03

**Authors:** Noelia Clemente Plaza, Manuel Reig García-Galbis, Rosa María Martínez-Espinosa

**Affiliations:** 1 Biochemistry and Molecular Biology Division, Department of Agrochemistry and Biochemistry, Faculty of Science, University of Alicante, 03690 San Vicente del Raspeig, Spain; noelia_noseque@hotmail.com; 2 Department of Nutrition and Dietetics, Faculty of Health Sciences, University of Atacama, Copiapó 2862, Chile; manuel.reig@uda.cl

**Keywords:** l-cysteine, biological medicine, nutraceutical, nutritional therapy, dietary supplements

## Abstract

This review summarizes recent knowledge about the use of the amino acid l-Cysteine (l-Cys) through diet, nutritional supplements or drugs with the aim to improve human health or treat certain diseases. Three databases (PubMed, Scopus, and Web of Science) and different keywords have been used to create a database of documents published between 1950 and 2017 in scientific journals in English or Spanish. A total of 60,885 primary publications were ultimately selected to compile accurate information about the use of l-Cys in medicine and nutritional therapies and to identify the reported benefits of l-Cys on human health. The number of publications about the use of l-Cys for these purposes has increased significantly during the last two decades. This increase seems to be closely related to the rise of nutraceutical industries and personalized medicine. The main evidence reporting benefits of l-Cys usage is summarized. However, the lack of accurate information and studies based on clinical trials hampers consensus among authors. Thus, the debate about the role and effectiveness of supplements/drugs containing l-Cys is still open.

## 1. Introduction

**Figure 1:**
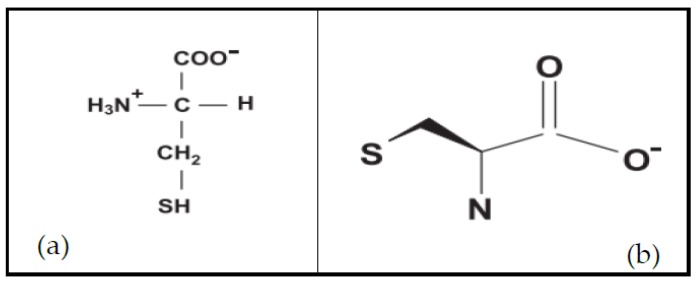
Chemical structure (**a**) and structural model (**b**) of an l-cysteine molecule (adapted from [1]).

l-cysteine (l-Cys) is a non-essential amino acid and thus is one of the building blocks required for the synthesis of proteins. It contains sulfur in the form of a thiol group (-SH) at the end of its side chain [1] (Figure 1). The -SH group is responsible for the high reactive capacity of the amino acid, and therefore is responsible for many of its biological functions in human beings. l-Cys is the amino acid establishing disulfide bridges, a type of covalent bond that plays a fundamental role in the folding and stabilization of the tertiary structure of the proteins, thereby supporting their biological activities [2,3]. The presence of conserved Cys in protein motifs found in all organisms could indicate that this feature was harnessed in early evolution to support enzyme catalysis, transcriptional regulation, protein folding, and 3-dimensional structure [4,5].

Cys is synthesized from methionine (an essential amino acid) thanks to two chemical reactions [3]. The first of these reactions is a transmethylation reaction, from which homocysteine is obtained as product. Then, homocysteine is transformed into cysteine through a transsulfuration reaction [1,6].

**Figure 2:**
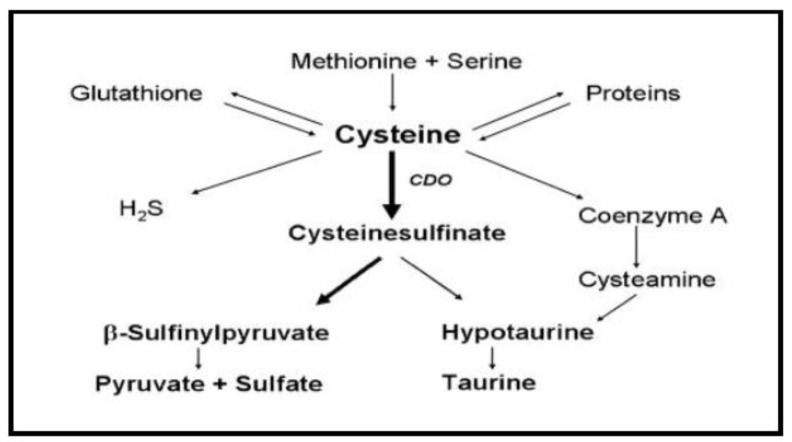
Summary of cysteine metabolism [8].

Cysteine can be assimilated through different pathways depending on the needs of the cells, giving rise to sulfur compounds [6,7]. The main metabolite obtained by the assimilation of Cys is sulfinate, a molecule that is metabolized to give rise to sulfinylpyruvate and pyruvate, or hipotaurine and taurine (Figure 2). On the one hand, taurine is a fairly abundant molecule at the intracellular level. Although the biological role of taurine is not at all clear, some studies suggest that it may be a compound involved in the nervous system, being especially necessary for brain development [8]. This suggestion is supported by the fact that high levels of taurine have been found in the fetal brain [9]. In addition, taurine displays many other biological roles, such as the marking of certain toxic intermediaries or the regulation of intracellular calcium levels. On the other hand, the sulfinylpyruvate produced during metabolism of Cys may undergo oxidation to sulfate, which is later used in the synthesis of 3′-phosphoadenosine-5′-phosphosulfate. There are many other important reactions occurring during the metabolism of Cys that allow the synthesis of thiocysteine or the transfer of sulfur between molecules [1].

The human genome encodes about 214,000 Cys-coding sequences [4]. Thus, the presence of this amino acid in proteins is significant. The reactivity and diverse functions of Cys are mirrored by a spectrum of susceptibilities and dysfunctions of their respective proteins, resulting in central roles for the Cys proteome in development, signal transduction, biologic defenses, aging, and disease [4,5,10]. Due to these important implications, l-Cys has attracted much attention of medical researchers and professionals worldwide (mainly in the USA and Europe).

Over the past few years, important biological functions of l-Cys in human beings have been described for the first time [4,5,6,11,12]. At the same time, the potential applications of this compound at the industrial level have been increasing, mainly in fields related to pharmaceuticals, medicines, and nutraceuticals. As examples of these applications, l-Cys plays an important role in the food industry (it is used as flavoring or as a chelating agent) [13], pharmaceutical industry (it is part of several drug formulas, for instance in drugs used to reduce the levels of acetaldehyde in the oral cavity) [14] and the cosmetics industry (l-Cys as part of skin or hair care formulas) [15,16].

Medicines and pharmaceuticals based on natural products are currently in an important period of expansion worldwide. Not only patients but also professionals in medicines, pharmaceuticals, and cosmetics look for new formulas and less aggressive therapies, in many cases integrating compounds/drugs of biological origin [17]. Thus, “personalized medicine”, based on drugs of natural origin as well as clinical nutrition, has experienced a boom in Western countries during the last few decades [17]. Besides, advances in biotechnology have promoted the design of a huge number of drugs and supplements for the prevention and the treatment of various diseases [17,18].

l-Cys is a compound widely used in the development of numerous drugs so far. However, the number of clinical trials testing the effects of l-Cys on human health and wellness is currently scarce. So, the impact of l-Cys usage (as part of drugs or as dietary supplements) on human health is a controversial issue.

This original work is based on a systematic review of the scientific studies published on l-Cys using different quality criteria to select the accurate and useful information [19]. The analysis presented here sheds light on the role and potential positive effects of its use in the prevention and treatment of diseases, as well as in the improvement and maintenance of health status.

## 2. Objectives

The main objective is to carry out an exhaustive, accurate, and rigorous analysis of the information available in scientific databases to identify the effects of the usage of l-Cys in human health. The analysis considers the use/administration of l-Cys as part of the formulations of drugs, nutraceuticals, or through food supplementation. This objective can be divided into two sub-objectives: (1) To compile information about the use of l-Cys in medicines and nutritional therapies (including clinical trials); (2) To identify the benefits reported by l-Cys on human health.

## 3. Results

### 3.1. Compilation of Bibliographic Sources of Interest

After a general search through Google Scholar, the major search engines PubMed, Scopus, and Web of Science were used to compile information about l-Cys available up to December 2017. This search indicated that the number of original papers identified through PubMed was significantly higher than the other two databases mentioned. Consequently, PubMed was the database ultimately chosen to identify and select the documents of interest for this systematic review. A total of 126,919 studies were thereby identified using the keyword “l-cysteine” (122,195 of them in English).

**Figure 3:**
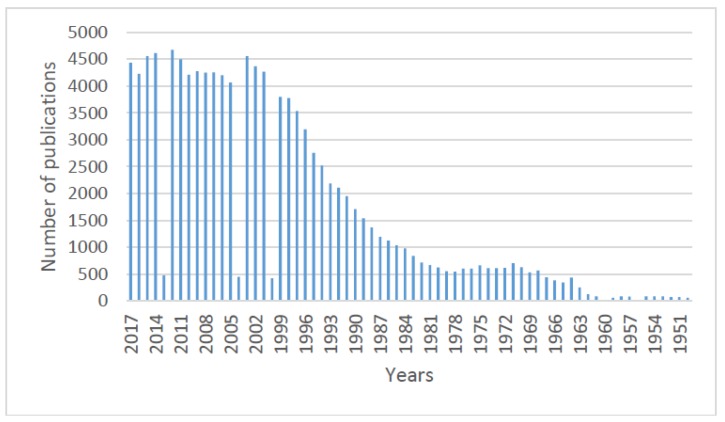
Number of publications per year (January 1950–December 2017). Database: PubMed. Keyword: l-Cysteine.

The analysis of the documents reveals that the earliest publications focusing on l-Cys can be traced back to the first third of the 20th century. Nevertheless, the number of scientific publications concerning l-Cys has mainly increased during the last four decades (Figure 3).

In total, 92% of the identified studies were descriptions of the physico-chemical properties of l-Cys or its biological roles. From the total, 8304 (6.54%) studies correspond to systematic reviews and 1638 (1.29%) are clinical trials. A total of 60,885 primary publications (47.97% of the total identified) were ultimately selected to comprise the database used in this research. These publications were selected considering the selection and exclusion criteria indicated in the materials and methods section, and combining the previous keyword (l-cysteine) with other terms of interest for this study (“l-cysteine & human health”; “l-cysteine & pharmacology”; “l-cysteine & food processing”; “l-cysteine & nutritional therapy”; “l-cysteine & nutrition”) (Table 1).

### 3.2. Bibliometric and Bibliographic Analysis

The increasing interest worldwide for natural compounds and their application in medicines, cosmetics, pharmaceuticals, and nutrition has impacted l-Cys research too. As an example, it is important to highlight that the number of papers reporting positive results from the usage of l-Cys in medicines and nutritional therapies has significantly increased during the last decade. As is displayed in Table 1, the United States and United Kingdom are the main countries from which the selected contributions originate (about 30% each), followed by Japan. Italy, France, and Spain are the European countries with the highest number of studies in this field at the time of writing this manuscript.

The bibliometric analysis of the selected information states that the greatest number of scientific–technical publications were within the field of pharmacology (52,873) (Table 1). It is very striking that, despite the great scientific and technical production on l-Cys and its potential uses, there is no author/institution and/or journal that significantly highlights the topic. Therefore, it could be concluded that it is a topic of great interest analyzed from very different perspectives and for multiple applications.

The increase in the number of publications about l-Cys during the last two decades is meaningful and relevant (Figure 3). To understand it, some reflections must be made about the recent boom in “personalized medicine” globally as well as about nutritional habits in developed countries. Interest in personalized medicine increased significantly between the end of the 20th century and the beginning of the 21st century as a result of [19]: (i) a great discontent with traditional treatments in certain segments of the population (some traditional treatments in occidental and oriental medicine are unable to cure or relieve symptoms in many cases); (ii) personalized treatments being highly demanded in developed countries; and (iii) the rejection of the population regarding chemically synthesized drugs becoming significant. In addition, the recognition of nutrition as a key factor for maintaining and restoring health has also contributed to the general interest for compounds of natural origin in general, and for l-Cys and other amino acids in particular.

### 3.3. Review of Clinical Trials on Effects of l-Cysteine on Human Health

Special attention has been paid to research based on recently published clinical trials to identify the possible advantages and disadvantages arising from the use of this amino acid in the context of medicines and nutritional supplements. A total of 1638 clinical trials were selected using the keyword combinations (Table 1). In total, 1431 of these studies showed significant evidence (both negative and positive) on the uses of the l-Cys alone or in combination with other compounds such as vitamin D or glycine. To make the reading of this work more understandable, the benefits of the usage of l-Cys in human beings have been summarized and classified into two tables as follows: Table 2 summarizes the main effects of the usage of l-Cys (alone) on human beings; Table 3 displays the benefits of its usage for human beings when the amino acid is combined with other molecules such as vitamin D or glycine. Comments on negative effects of the usage of l-Cys are also summarized after Table 2.

Regarding the use of l-Cys as part of drug formulas or nutritional supplements, the following benefits have been reported (Table 2): antioxidant power, regulation of the mucolytic function, strengthening of the hair, improvement of the functions of the immune system, protection and detoxification of the liver, promotion or elimination of heavy metals, prevention of heart disease, diabetes prevention, delay of aging, and the protection of the digestive system.

Some studies suggest that there is no clear effect (neither positive nor negative) of l-Cys on human health whilst some recent works state that l-Cys could behave as a negative modulator on GABAergic neurotransmission [20].

**Figure 4:**
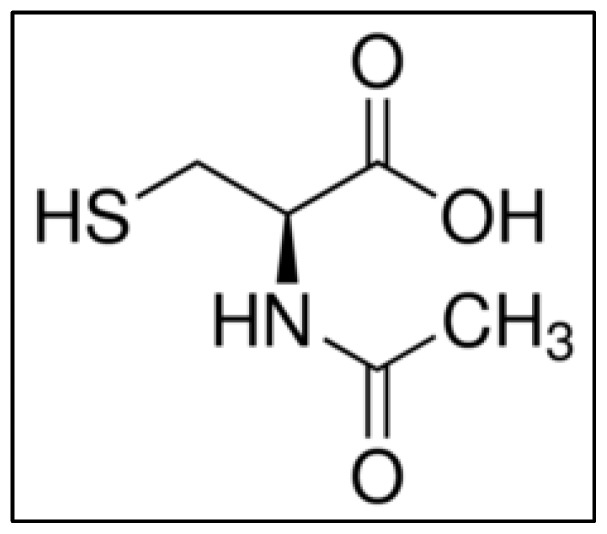
Chemical structure of *N*-acetyl-l-cysteine (NAC).

Although some prominent clinical trials have tested the effects of l-Cys, most of them have analyzed the effects of *N*-acetyl-l-cysteine (NAC) instead [39,40]. This compound (Figure 4) is a precursor of l-Cys that promotes glutathione biosynthesis [41]. It acts directly as a scavenger of free radicals, mainly oxygen radicals. Consequently, it is a powerful antioxidant useful for treating several disorders that result from the generation of free oxygen radicals. Additionally, it is a highly efficient mucolytic drug promoting tenacious mucous discharges [42]. The main effects of its usage in human beings are summarized in Table 4.

In brief, NAC supplementation has exerted favorable effects on vascular health, muscle strength, bone density, cell-mediated immunity, preservation of cognitive function, or marking systemic inflammation [49,67,69,70,71,72]. Although positive effects of NAC have been reported for treatments of patients with acute liver failure [61], other studies on acute liver failure and liver surgery suggest patients randomized to postoperative NAC received no benefit [62,63,64]. Finally, NAC is part of the paracetamol formula (acetaminophen). When paracetamol is taken in large quantities, a minor metabolite called *N*-acetyl-*p*-benzoquinone imine (NAPQI) accumulates within the body. It is normally conjugated by glutathione, but when taken in excess, the body’s glutathione reserves are not sufficient to deactivate the toxic NAPQI. This metabolite is then free to react with key hepatic enzymes, thereby damaging liver cells. This may lead to severe liver damage and even death by acute liver failure. This phenomenon is commonly named paracetamol poisoning [73,74,75].

## 4. Discussion

The review of all selected documents, and particularly the clinical trials, has enabled identification of the effects of the usage of l-Cys in human health and wellness, either by its application individually or combined with other compounds. The clearly demonstrated benefits on human health are as follows:

- Antioxidant role. l-Cys acts as a precursor for the synthesis of glutathione, which is an important antioxidant. The reduced form of glutathione plays a fundamental role in the defense of the organism against damage caused by oxidative stress [76]. This property is due to its ability to neutralize reactive particles that can cause damage to cells and tissues. Thus, diet supplementation with l-Cys restores the synthesis of glutathione in cases in which it has been compromised, thus improving the redox balance and promoting the reduction of oxidative stress. In addition, the elimination of free radicals may also be associated with certain benefits, such as in the case of reduced healing time following certain surgical procedures (photorefractive keratectomy, for instance) [22]. Besides, the antioxidant role of l-Cys is also related to reduced risk of a cerebrovascular accident [23] and reduced noise-induced hearing loss [66].
- Mucolytic function. NAC causes a decrease in the viscosity of mucus secretion, thus facilitating their elimination. Bronchial secretions contain high concentrations of mucoproteins. The decrease in viscosity promoted by NAC is mainly due to the breakage of the disulfide bonds of the mucoproteins, resulting in a fragmentation of the chains from the mucins, immunoglobulins and serum albumin present in the mucous secretion [40].
- Strengthening of hair. Blends fortified with l-Cys help to strengthen hair. Keratin is one of the most abundant proteins in the skin and the hair, and contains high amounts of l-Cys as building blocks. l-Cys forms disulfide bridges, which provide strength and rigidity to keratin. Consequently, the use of blends fortified with l-Cys promotes the repair of structural lesions and slows down hair loss experienced by patients affected by certain disorders (diffuse alopecia, for instance [24]).
- Regulation of the activity of the immune system. It has been reported that l-Cys can regulate immune system activity by promoting changes in levels of production of its effector molecules, as is the case of IL-17. IL-17 is a cytokine produced mainly by some T-cells, called Th17 cells, which act on epithelial cells and fibroblasts, as well as on other cells of the immune system. It has also been demonstrated that the administration of NAC can significantly improve transplant-free survival in patients affected by acute liver failure not related to acetaminophen when administered during the early stages of hepatic encephalopathy. This effect is due to the regulation of IL-17 production, which is closely related to the progression of the encephalopathy [61].
- Protection of the digestive system. Excessive alcohol consumption is considered a risk factor for the development of cancer in the upper gastrointestinal tract, because of exposure to acetaldehyde, which is carcinogenic to humans. In this sense, l-Cys intake reduces the concentration of acetaldehyde in saliva, thus decreasing the exposure of the gastrointestinal tract to this compound and, consequently, the risk of cancer [27].
- Reducing the risk of stroke. Some studies suggest that consumption of certain amino acids, among which l-Cys, may be related to certain cardiovascular benefits, such as reduced arterial stiffness or reduced blood pressure, thereby fighting some risk factors related to vascular accidents in healthy women [55].

Despite the high volume of evidence describing positive impacts of l-Cys on human health, it is worth noting that the number of works describing no effects or negative effects is also significant [20,77,78]. Negative effects of l-Cys derivatives on human health have been reported [79,80]. Thus, although most of the studies on l-Cys highlight its role in the homeostasis of redox status, some studies suggest that redox modulation is not involved during l-Cys actions and that l-Cys might act as a competitive antagonist of GABAA ρ1 receptors for instance [20]. Also, some in vivo studies have shown that several S-conjugates are nephrotoxic and that the toxicity is associated with β-lyase-dependent bioactivation [79]; in other cases, toxic effects of l-Cys on the nervous system have also been reported (oxidized l-Cys derivatives or compounds such as cysteine alpha-carbamate caused neuronal degeneration) [78].

So, the controversy surrounding the benefits of supplements or drugs containing l-Cys is still open, mainly regarding their use for some specific treatments. In fact, many of the studies have been done in vitro and translational studies are still scarce. The following considerations can be underlined, taking into account this controversy and the results obtained from this review:

(i) According to the results analyzed here, NAC is preferentially used instead of l-Cys. Besides, l-Cys is usually administrated as part of a formula containing other compounds, such as glycine, vitamin D, bFCF or theanine. So, the effects reported in these studies cannot be directly attributed solely to the l-Cys molecule.
(ii) Most of the studies done at the time of writing this review are based on laboratory tests and cellular lines.
(iii) The number of studies on the effects of l-Cys on human health based on clinical trials is limited and in many cases these are very preliminary studies in which the studied population does not reflect a standard population (considering aspects such as age, sex, etc.).
(iv) Several important details related to l-Cys and NAC doses and metabolism remain unknown. Optimal l-Cys doses and safety concentration ranges according to some pathologies are far from known. In addition, optimization of the assimilation of l-Cys and its derivatives, when applied through nutritional supplements, is poorly described.

## 5. Materials and Methods

### 5.1. Search Strategy and Information Processing

First, several searches of generic character in the “Google Scholar” portal (https://scholar.google.es/) were carried out. In this search, several important secondary sources were identified which have been helpful for the design of this systematic review and for writing the introduction section.

The second step in this work was the realization of a comprehensive bibliometric/bibliographic review through the major search engines available, PubMed, Scopus, and Web of Science (which are connected to specialized databases on the subject covered by this study). This search indicated that the number of original papers identified through PubMed was significantly higher than the other two databases mentioned. Consequently, PubMed was the database ultimately chosen to identify and select the documents of interest for this work. The search date was between January 1950 and December 2017 and included texts published in scientific journals in English and Spanish. The research questions were as follows: What role does l-cysteine play in pharmacology, human health, nutrition, and food processing? What is the effect of l-Cys on human health?

The information retrieval system “boolean” was used to identify the works of interest for this review [81]. The keywords used were as follows: “l-cysteine”; “l-cysteine & human health”; “l-cysteine & pharmacology”; “l-cysteine & food processing”; “l-cysteine & nutritional therapy”; “l-cysteine & nutrition”. These keywords were previously identified through the “MeSH” (Medical Subject Heading) database as descriptors for the realization of this work. The advanced search form was used in the PubMed database to identify the documents of interest. Additionally, the following options were selected: “Title/abstract”, “article”, “review”.

At a more advanced stage of the study, special attention was paid to clinical trials (most of them published over the last 25 years) to identify the main uses and possible effects of l-Cys in its application within the field of medicine.

### 5.2. Data Extraction and Selection of Relevant Studies

A total of 126,919 articles were identified but 60,885 were ultimately selected to constitute the database for this research. The selection criteria were as follows: primary sources, review/original articles, clinical trials, and studies with objective data on direct correlations between the use of l-Cys and effects on human health (adults, adolescents, and children). In all the selected studies, the direct correlations were established when l-Cys was naturally part of the food intake or as part of nutritional supplements. The exclusion criteria were as follows: articles of non-specific systematic review, meta-analyses of humans, or other non-human clinical trials with non-representative sample size. Each of the selected articles has been analyzed by the three authors with backgrounds in biology, biochemistry and nutrition. The classic scheme proposed by Vilanova has been used to assure the quality of the selection criteria [19]. Thus, the following questions were considered to evaluate each of the identified works (quality criteria): is it related to the research objectives of this review? Is the methodology clear and objective? Is the study 100% reproducible? Is the sample size coherent? Is the sample well-defined? Is the sample representative? Is the hypothesis clearly stated?

## 6. Conclusions

Over the past few years, several benefits have been attributed to l-Cys and NAC, contributing to their becoming useful compounds within the food, pharmaceuticals, medicines or cosmetics industries. A considerable number of cysteine-rich products that are mainly used in hair and skin care have been commercialized. More recently, l-Cys has emerged as an important molecule in the food supplements industry, a sector that is in full growth, possibly due to increasingly widespread concern in the populations of developed countries to address nutritional deficiencies caused by new habits of life and the inability, on certain occasions, to follow a balanced diet. Thus, nutritional therapy and functional foods have involved l-Cys as part of treatment of several pathologies such as cirrhosis (in which l-Cys biosynthesis is compromised) [82], or simply to prevent cancer or promote good health [83,84]. Although several potential benefits have been attributed to l-Cys or NAC, only a few of these benefits have been clearly demonstrated. Some of these examples are (i) the use of these molecules as a mucolytic expectorant; (ii) positive effects on cirrhosis treatment or positive contributions in the maintenance of redox homeostasis in cells and in cancer treatment (through GSH synthesis) [85,86]. Another important aspect to point out is the potential use of l-Cys in processes related to food conservation and processing. l-Cys shows antioxidant, chelating, and flavoring properties which could be useful in food industries [13,87]. However, few applications of this amino acid are already available on the market (and only in developed countries).

In summary, although the available literature on l-Cys and NAC is abundant, several key questions remain unaddressed. Consequently, more effort must be made in the near future to establish clear and direct connections between these two molecules and benefits on human health. These connections should be made not only based on in vitro analysis but also based on clinical trials carried out under quality standards and good practices as has been recently recommended [88,89].

## Figures and Tables

**Table 1:** Number of publications included in the database for this work (Database: PubMed). * %: calculated over the total number of publications compiled from each combination of keywords.

Keywords	Total of Publications	Publications in the Last 5 Years (%)*
l-cysteine & pharmacology	52,873	9976 (19%)
l-cysteine & human health	4742	1725 (36%)
l-cysteine & nutrition	1891	579 (31%)
l-cysteine & nutritional therapy	1074	265 (25%)
l-cysteine & food processing	305	127 (42%)

**Table 2:** Summary of the potential effects of l-Cys (alone) supported by studies based on clinical trials.

Examples of l-Cysteine Usage	Effects	Ref.
Nutritional therapy in children with severe edematous malnutrition	Restoration of the rate of synthesis and the concentration of glutathione during the first phase of treatment	[21]
Scarring of the cornea after a photoreactive keratectomy	Reduced average time of scarring	[22]
Nutritional therapy in Ictus patients	Reduced risk of cardiovascular accident	[23]
Hair care	Reduced hair loss and increased hair strengthening abilities	[16,24]
Protection of digestive system	Reduction in the concentration of acetaldehyde by avoiding exposure in cases of achlorhydria	[25]
Treatment chronic inflammation	Increased antioxidant status	[26]
Prevention of upper digestive tract cancer and breast cancer	Decrease of acetaldehyde in saliva or it can be used as part of metabolic starvation therapy	[27,28]
Indicator for the control of cardiovascular diseases	Pro-inflammatory signaling	[29]
Treatment of erythropoietic porphyria	Photosensitivity improvement	[30]
Treatment of type-2 diabetes	Control of glycaemia and vascular inflammation	[31,32,33]

**Table 3:** Summary of the potential effects of l-Cys (combined with other compounds) supported by studies based on clinical trials.

Composition of the Mixture	Examples of Usage	Effects	Ref.
l-Cysteine + Glycine	Nutritional therapy in elderly HIV + patients	Improved oxidation of carbohydrates, insulin sensitivity and body composition	[34,35]
	Treatment of oxidative stress during aging	Increased synthesis of glutathione and decreases oxidative stress levels	
l-Cysteine + glycine + dithreonine	Treatment of hypostatic ulcer	Reduced pain and improved degree of ulcer healing	[36]
l-Cysteine + vitamin D	Treatment of patients with type-2 diabetes	Increased levels of glutathione and decreased levels of triglycerides	[33]
l-Cysteine + basic fibroblast growth factor (bFCF)	Treatment of corneal epithelium after photoreactive keratectomy in patients affected by myopia	Reduced time of resurfacing corneal.	[37]
l-Cysteine + theanine	Improvement of well-trained athletes’ performance	Restoration of the attenuation of the activity of Natural Killer cells	[38]

**Table 4:** Summary of the potential effects of NAC supported by studies based on clinical trials.

Examples of usage	Effects	Ref.
Treatment of methamphetamine-dependent patients	Methamphetamine dependence decreases	[43]
Performance of athletes undergoing strenuous physical training	Redox equilibrium and adaptation processes improve	[44]
Treatment of Thalassemia	Oxidative stress and DNA damage decrease	[45,46]
Protection against the carcinogenic effect of tobacco	Modulation of biomarkers associated with cancer	[47]
Treatment of bacterial meningitis	Antioxidant role	[48,49]
Treatment against influenza virus	Proliferation of the virus is inhibited	[49,50]
Mucolytic expectorant and treatment of respiratory tract infections	The viscosity decreases and facilitates the removal of mucus	[40,51]
Specific antidote for acetaminophen overdose	Regeneration of glutathione levels	[49,52,53,54]
Cardiovascular complications in patients with diabetes Treatment of type-2 diabetes	Attenuation of cardiovascular complications	[55,56,57]
Prevention of cardiovascular diseases	Reduction of plasma concentrations and homocysteine levels	[58]
Treatment of chronic hepatitis C	Increase in glutathione and improvement in response to treatment with interferon	[59,60]
Treatment of patients with acute liver failure	Reduced IL-17 levels	[61,62,63,64]
Treatment of nephropathic cystinosis	Reduced oxidative stress and improved renal function	[65]
Treatment of noise-induced hearing loss	Protective effect. Hearing loss is reduced	[66]
Treatment of cocaine addiction	It acts as an anti-relapse agent in abstinent subjects	[67,68]

## Abbreviations

bFCF	Basic fibroblast growth factor
IL-17	Interleukin 17
l-Cys	l-cysteine
NAC	*N*-acetyl-l-cysteine
PAPS	3′-phosphoadenosine-5′-phosphosulfate
